# A disintegrin and metalloproteinase 8 induced epithelial‐mesenchymal transition to promote the invasion of colon cancer cells via TGF‐β/Smad2/3 signalling pathway

**DOI:** 10.1111/jcmm.15907

**Published:** 2020-09-20

**Authors:** Qianna Jin, Xin Jin, Tao Liu, Xiaoming Lu, Guobin Wang, Nan He

**Affiliations:** ^1^ Department of Radiology Union Hospital Tongji Medical College Huazhong University of Science and Technology Wuhan China; ^2^ Cancer Center Union Hospital Tongji Medical College Huazhong University of Science and Technology Wuhan China; ^3^ Department of Gastrointestinal Surgery Union Hospital Tongji Medical College Huazhong University of Science and Technology Wuhan China

**Keywords:** ADAM8, colon cancer, EMT, Smad, TGF‐β

## Abstract

A disintegrin and metalloproteinase 8 (ADAM8) protein is a multi‐domain transmembrane glycoprotein which involves in extracellular matrix remodelling, cell adhesion, invasion and migration. ADAM8 and epithelial‐mesenchymal transition (EMT) play an important role in tumour invasion has been well established. However, the interaction between ADAM8 and EMT has remained unclear. The data of colon cancer patients obtained from TCGA (The Cancer Genome Atlas) and GTEx (Genotype‐Tissue Expression Project) were analysed by the bioinformatics research method. The expression of ADAM8 in colon cancer cells was up‐regulated and down‐regulated by transfecting with the expression plasmid and small interfering RNA, respectively. Transwell invasion assay, immunohistochemistry, immunocytochemistry, Western blotting and qRT‐PCR were utilized to study the effect of ADAM8 on colon cancer cell's EMT and its related mechanisms. Analysis of TCGA and GTEx data revealed that ADAM8 was linked to poor overall survival in colon cancer patients. Besides, ADAM8 was correlated with multiple EMT biomarkers (E‐cadherin, N‐cadherin, Vimentin, Snail2 and ZEB2). In vitro, we also proved that the up‐regulation of ADAM8 could promote EMT effect and enhance the invasive ability of colon cancer cells. On the contrary, the down‐regulation of ADAM8 in colon cancer cells attenuated these effects above. Further studies suggested that ADAM8 modulated EMT on colon cancer cells through TGF‐β/Smad2/3 signalling pathway. Our research suggested that ADAM8 could be a potential biomarker for the prognosis of colon cancer and induced EMT to promote the invasion of colon cancer cells via activating TGF‐β/Smad2/3 signalling pathway.

## 1 INTRODUCTION

Colon cancer is a common digestive tract malignant tumour with high morbidity and mortality, which is a serious threat to human health.^1^ Although many different tumour biomarkers and treatments have emerged in recent years, there has been no obvious improvement in overall survival of colon cancer patients. Metastasis and recurrence are the main causes of death in colon cancer patients.^2^ Therefore, it is very urgent to find more effective tumour biomarkers and therapeutic targets. Epithelial‐mesenchymal transition (EMT) is crucial for the promotion of cell metastasis in malignant tumours. It is marked by the loss of epithelial cell characteristics and the acquisition of interstitial cell characteristics.^3^ In EMT, polar epithelium cells could be converted into mesenchymal cells which move freely between the matrices. Extensive researches have shown that once colon cancer cells develop EMT, their ability to invade and metastasize will be greatly enhanced.^4^, ^5^, ^6^

ADAM8, as one of the most important members of the ADAM family, has attracted increasing attention due to its close relationship with tumorigenesis, development and prognosis. ADAM8 is a multi‐domain transmembrane glycoprotein which involves in extracellular matrix remodelling, cell‐cell interactions and various aspects of carcinogenesis. Several studies have shown that ADAM8 is highly expressed in a variety of malignant tumour tissues, such as colon cancer, glioma, lung cancer, liver cancer, pancreatic cancer and gastric cancer, and promotes tumour invasion and metastasis.^7^, ^8^, ^9^, ^10^, ^11^, ^12^, ^13^, ^14^

While it is known ADAM8 and EMT play important roles in tumour invasion and metastasis, no prior studies have investigated the correlation between ADAM8 and EMT. In the present study, by analysing human colon cancer's data from TCGA and GTEx databases, we not only demonstrated that ADAM8 was closely related to poor prognosis in patients with colon cancer, but also found the correlation between ADAM8 and EMT‐related biomarkers. In addition, we found that ADAM8 could induce EMT to promote colon cancer cell invasion via activating TGF‐β/Smad2/3 signalling pathway. The importance and innovation of this study are to explore the correlation between ADAM8 and EMT in colon cancer cells and to highlight the pivotal role of ADAM8 in colon cancer cells’ invasion and EMT. Therefore, these findings will contribute significantly to the determination of ADAM8 as an effective biomarker and therapeutic target for colon cancer.

## 2 MATERIALS AND METHODS

### 2.1 Public data analysis

All the work on colon cancer's data from TCGA and GTEx database was carried out by using the online tool Gene Expression Profiling Interactive Analysis (GEPIA, http://gepia.cancerpku.cn/index.html).^15^ GEPIA performed survival analysis on gene expression levels and required a log‐rank test for hypothesis assessment. GEPIA also performed paired gene Pearson correlation analysis between ADAM8 and EMT‐related biomarkers by using colon cancer's data from TCGA and/or GTEx database.

### 2.2 Cell culture and tissue specimens

Six human colon cancer cell lines, including SW480, SW620, DLD1, HCT116, HCT8 and HT29, were purchased from the cell bank of Chinese Academy of Science. Four cell lines of them (SW480, SW620, DLD1 and HCT8) were cultured in RPMI 1640 (HyClone) supplementing with 10% foetal bovine serum (FBS; HyClone). The other 2 cell lines (HCT116 and HT29) were cultured in McCoy's 5A medium (Gibco) containing 10% FBS (HyClone). All cells were grown in a 37°C humidified incubator with 5% CO_2_. All tissue specimens, including colon cancer tissues and adjacent normal mucosa tissues (more than 5 cm from the edge of tumour), were derived from the department of general surgery at Wuhan Union Hospital from September 2016 to July 2017. Each specimen was divided into two parts, one stored in liquid nitrogen for qRT‐PCR testing and the other fixed in 4% formaldehyde solution for histopathological examination.

This study was conducted by the Code of Ethics of the World Medical Association (Declaration of Helsinki) and approved by the ethics committee of Wuhan Union Hospital, China.^16^ Informed consent was issued by all patients in this study.

### 2.3 Stably transfected cell lines

HCT116 cells with lower endogenous ADAM8 expression levels (as shown in Figure 4A) were selected for transfecting with ADAM8 overexpression plasmid (Flag‐ADAM8; Sigma‐Aldrich) or empty vector (pCDNA3.1; Sigma‐Aldrich), and Lipofectamine 2000 (Invitrogen) used as the transfection reagent according to the manufacturer's protocol. After 24 hours, cells were treated with 600 mg/mL G418 (Invitrogen). The transfection efficiency on cell lines with ADAM8 overexpression plasmid or empty vector was evaluated by Western blotting and qRT‐PCR.

### 2.4 RNA interference

Small interfering RNA (siRNA) oligonucleotides which were specific to ADAM8 and Smad2/3 obtained from RiboBio Co., Ltd. SW480 cells with higher endogenous ADAM8 expression (as shown in Figure 4A) were cultured in six‐well plates until 60% confluence and transfected with 50 nmol/L of the indicated siRNA using riboFECT™ CP Reagent (RiboBio) according to the manufacturer's instructions. The efficiency of gene knockdown was analysed by Western blotting and qRT‐PCR after 48 hours.

### 2.5 Cell invasion assay

The invasive ability of tumour cells was detected by Transwell invasion assay. While medium supplemented with 10% FBS was placed in the lower chamber, 1 × 10^5^ cells were seeded in upper cell culture insert containing 8.0 μm pore size membrane (Corning) and cultured in FBS‐free medium. A diluted extracellular matrix gel (Corning) was coated on the membrane of cell culture insert for the invasion assay. Following incubation for 48 hours, the invaded cells below the membrane was fixed with 4% paraformaldehyde and stained with 0.1% crystal violet. The invaded cells were counted and photographed by using inverted microscope.

### 2.6 Western blotting

Total protein was isolated from the colon cancer cells and determined the concentration by using Coomassie protein assay (Thermo Fisher Scientific). Protein sample from the previous step was separated by SDS‐PAGE and transferred to polyvinylidene difluoride membrane. After blocking for 30 minutes in skim milk at room temperature, the membrane was incubated with specific primary antibody overnight at 4°C. The next day, the membrane was incubated with secondary antibody (1:5000 dilution, Cell Signaling Technology) for 2 hours at room temperature. GAPDH was served as a control. The signal on the membrane was detected and imaged by Odyssey infrared imaging system (LI‐COR). The expression of all protein was quantified by Image J software and normalized to the quantified value of GAPDH. These following antibodies were used in Western blotting experiments, such as ADAM8, Slug (Snail2), ZEB2 (1:1000, Abcam); E‐cadherin, α‐SMA, Vimentin, TGF‐β, Smad2, p‐smad2, Smad3, p‐smad3 and GAPDH (1:1000, Cell Signaling Technology).

### 2.7 TMA, IHC staining and evaluation

The Tissue microarray (TMAs) were constructed using an automated TMA instrument (ALPHELYS, Plaisir). Immunohistochemistry (IHC) staining was performed on the TMA slides. All tissue specimens were fixed in 4% formaldehyde solution, embedded into paraffin and cut in 4‐μm sheets. The tissue slides dewaxed with xylene, dehydrated by using gradient alcohol and retrieved antigen by microwave. ADAM8 antibody (Abcam) was diluted to 1:200. The secondary antibody was labelled with peroxidase and developed with diaminobenzidine. The staining procedure was carried out according to the manufacturer's instructions. The result of IHC was judged by two experienced pathologists and quantified by using a semi‐quantitative comprehensive scoring method which based on the intensity of staining and the ratio of stained cells: the immunoreactive score (IRS) depended on the intensity of staining (SI) and the percentage of positive cell (PP), IRS = SI+PP. SI: 0 points for the colourless, 1 point for the light yellow, 2 points for the brownish yellow and 3 points for the tan. PP: 0 points for negative, 1 point for positive cells ≤10%, 2 points for 11% to 50% and 3 points for 51% to 100%. IRS: 0 negative, ≤3 low expression and >3 high expression.^17^

### 2.8 Immunocytochemistry

5 × 10^5^ cells were seeded in a 24‐well plate with 12‐mm round glass coverslip. After 4% paraformaldehyde fixation, coverslips were washed three times with PBS, incubated in 0.4% Triton X‐100 at room temperature for 10 minutes, blocked in 5% BSA for 1 hour at room temperature with gentle shaking and incubated in Anti E‐cadherin antibody (1:400, Cell Signaling Technology) overnight at 4°C with gentle shaking. The next day, coverslips were washed with PBS and incubated for 1 hour at room temperature with anti‐rabbit Alexa‐Fluor 488 (1:400, Thermo Fisher Scientific). Finally, coverslips were washed with PBS and imaged by microscopy (Zeiss Axiostar plus with HBO 50 fluorescent lamp, Zeiss).

### 2.9 Chromatin immunoprecipitation (ChIP) and ChIP‐qPCR

ChIP was performed following the manufacturer's instructions for the Chromatin Extraction Kit (Abcam) and ChIP Kit Magnetic ‐ One Step (Abcam).^15^ Smad 2/3 (Cell Signaling Technology, dilution 1:100) was used for the ChIP assay. The purified DNA was analysed by real‐time PCR with a PCR kit (Takara) according to the manufacturer's protocols. The following primers were used: E‐cadherin ChIP primer, forward 5′‐ACTCCAGGCTAGAGGGTCACC‐3′ and reverse 5′‐CCGCAAGCTCACAGGTGCTTTGCAGTTCC‐3′. The ADAM8 inhibitor (BK‐1361) was purchased from Probechem Biochemicals Co., Ltd, USA.

### 2.10 RNA isolation and quantitative polymerase chain reaction

Total RNA extracted with TRIzol reagent (Invitrogen) according to the manufacturer's protocol. Complementary DNA was reverse‐transcribed using a reverse transcription kit (Takara). Quantitative reverse transcription‐polymerase chain reaction (qRT‐PCR) was performed with SYBR Green Master Mix Kit (Thermo Fisher Scientific) by using ABI 7500 System (Thermo Fisher Scientific). β‐actin used as a control for the normalization of gene expression. The 2^−∆∆CT^ method was utilized to quantify the fold change.^13^ All primers were synthesized by Sangon Biotechnology Co., Ltd and listed in Table 1.

**Table 1:** Quantitative RT‐PCR primer sequences

Gene	Forward (5′‐3′)	Reverse (5′‐3′)
ADAM8	ACAATGCAGAGTTCCAGATGC	GGACCACACGGAAGTTGAGTT
E‐Cadherin	GGCACAGATGGTGTGATTACAGTCAAAA	GTCCCAGGCGTAGACCAAGAAA
N‐cadherin	GACGGTTCGCCATCCAGAC	TCGATTGGTTTGACCACGG
Vimentin	GCTTCAGAGAGAGGAAGCCGAAAA	TTTCCAAGCCTGACCTCACGG
b‐actin	CCCTGGCTCCTAGCACCAT	AGAGCCACCAATCCACACAGA

### 2.11 Statistical analysis

Statistical analysis was performed with SPSS 25.0 (SPSS Inc) and Prism software (GraphPad). Pearson's chi‐squared test and Fisher's exact test were applied for categorical variables, and continuous variables were analysed by the Student's *t* test. All statistical analyses were two‐sided, and *P* < .05 was considered statistically significant. All values represent the means ± SD.

## 3 RESULTS

### 3.1 High level of ADAM8 associated with poor prognosis in colon cancer patients

**Figure 1:**
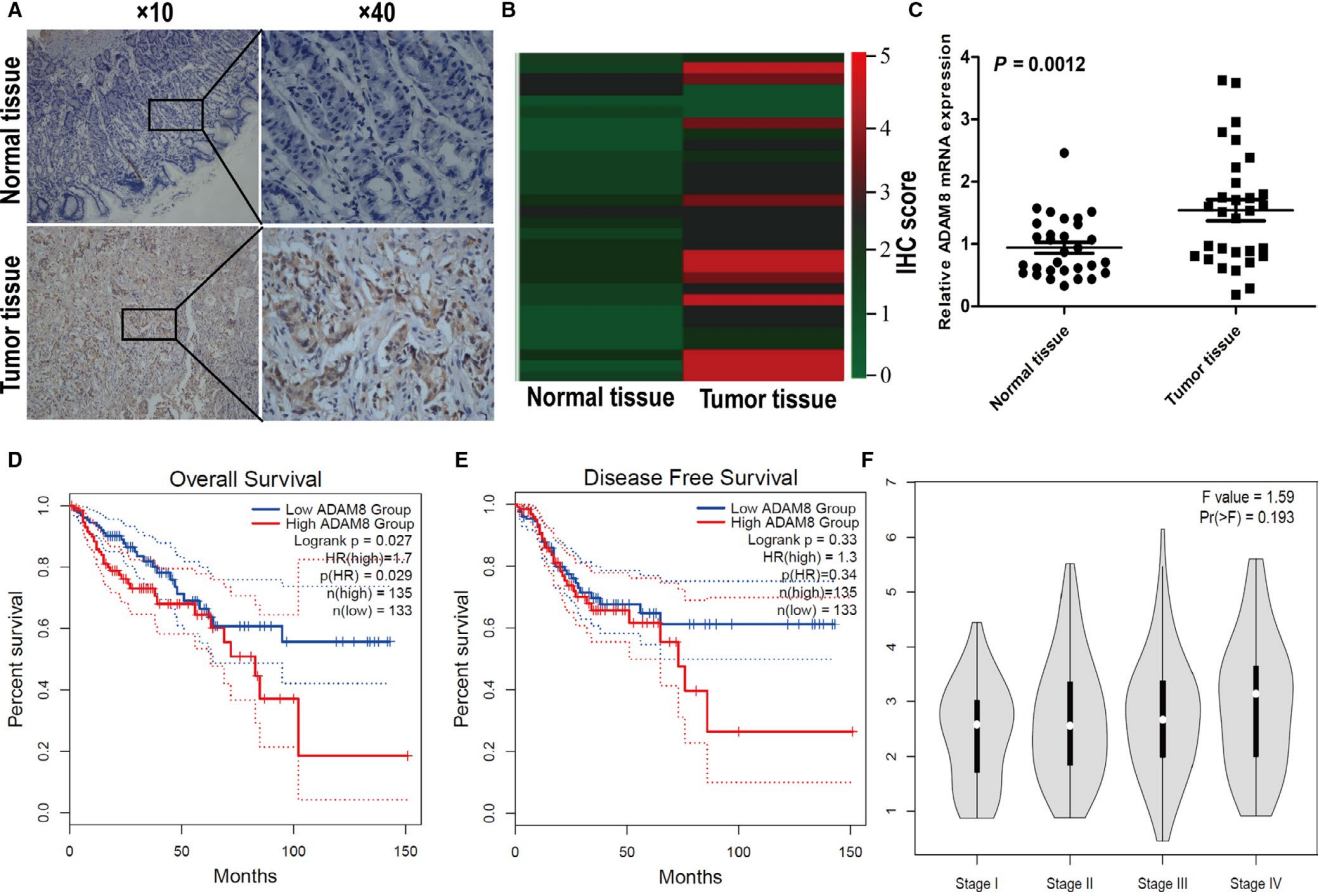
The level of ADAM8 associated with poor prognosis in colon cancer patients. A, Representative image of ADAM8 expression in colon cancer tissues was detected by IHC. Scale bars were indicated. B, Heat map showed the IHC staining score of ADAM8 protein in normal mucosal tissue and tumour tissues. C, Expression of ADAM8 mRNA detected by qRT‐PCR in normal mucosal tissue and tumour tissues. D, Overall survival and E, disease‐free survival, GEPIA performed survival analysis and required a log‐rank test for hypothesis assessment. F, The level of ADAM8 was presented in different tumour stages. ADAM8, a disintegrin and metalloprotease 8; mRNA, messenger RNA; qRT‐PCR, quantitative reverse transcription‐polymerase chain reaction; *P < .05; **P < .01; ***P < .001; ns, no significant

To evaluate the level of ADAM8 in colon cancer tissues and adjacent normal mucosa tissues, the expression of ADAM8 protein and mRNA was examined in a group of 30 colon cancer tissues paired with adjacent normal mucosa tissues by IHC and qRT‐PCR. IHC results revealed that specific ADAM8 staining was detected in the cytoplasm and cell membrane of epithelial cells (Figure 1A,B). The IHC staining scores of ADAM8 in colon cancer tissues were higher than that of adjacent normal mucosa tissues (3.13 ± 0.27 vs 1.17 ± 0.18, respectively; *P* < .001). Furthermore, the mRNA level of ADAM8 at cancer tissues was significantly higher than that of adjacent normal tissues (1.54 ± 0.17 vs 0.94 ± 0.08, respectively; *P* = .0012; Figure 1C).

The online tool GEPIA was applied to analyse colon cancer patients’ data from TCGA and GTEx databases. The results showed that patients with high ADAM8 expression had poorer overall survival than patients with low ADAM8 expression (*P* = .029, HR = 1.7; Figure 1D). There were no significant differences in disease‐free survival (*P* = .33, HR = 1.3; Figure 1E) and no correlation between the level of ADAM8 and the tumour pathological stage (*F* = 1.59, Pr = 0.193; Figure 1F). However, in our validation cohort, we performed IHC staining assay in TMAs of 97 colon cancer samples. We pooled the level of ADAM8 with patients’ clinical‐pathologic factors for statistical analysis. The results showed that the level of ADAM8 in colon cancer was significantly related to patients’ AJCC stage, depth of invasion, N stage and distant metastasis (*P* < .05; Table 2).

**Table 2:** Association between ADAM8 expression and patient clinical‐pathologic characteristics in colon cancer patients

Factors	n	ADAM8 expression		*P* value
		High	Low	
Age, y				.160
<65	44	22	22	
≥65	53	34	19	
Gender				.174
Male	73	45	28	
Female	24	11	13	
Tumour size (cm)				.488
≤5	38	24	14	
>5	59	32	27	
Tumour location				.534
Right	39	24	15	
Others	58	32	26	
Differentiation				.824
Well	13	8	5	
Moderate	26	16	10	
Poor	58	32	26	
AJCC stage				.001
I + II	33	11	22	
III + IV	64	45	19	
T stage				.014
T1 + T2	29	11	18	
T3 + T4	68	45	23	
N stage				.007
N0	18	5	13	
N1 + N2	79	51	28	
M stage				.02
M0	90	49	41	
M1	7	7	0	

Chi‐squared test and Fisher's exact test were applied for categorical variables, and continuous variables were analysed by the Student's *t* test.

### 3.2 ADAM8 was related to EMT biomarkers

**Figure 2:**
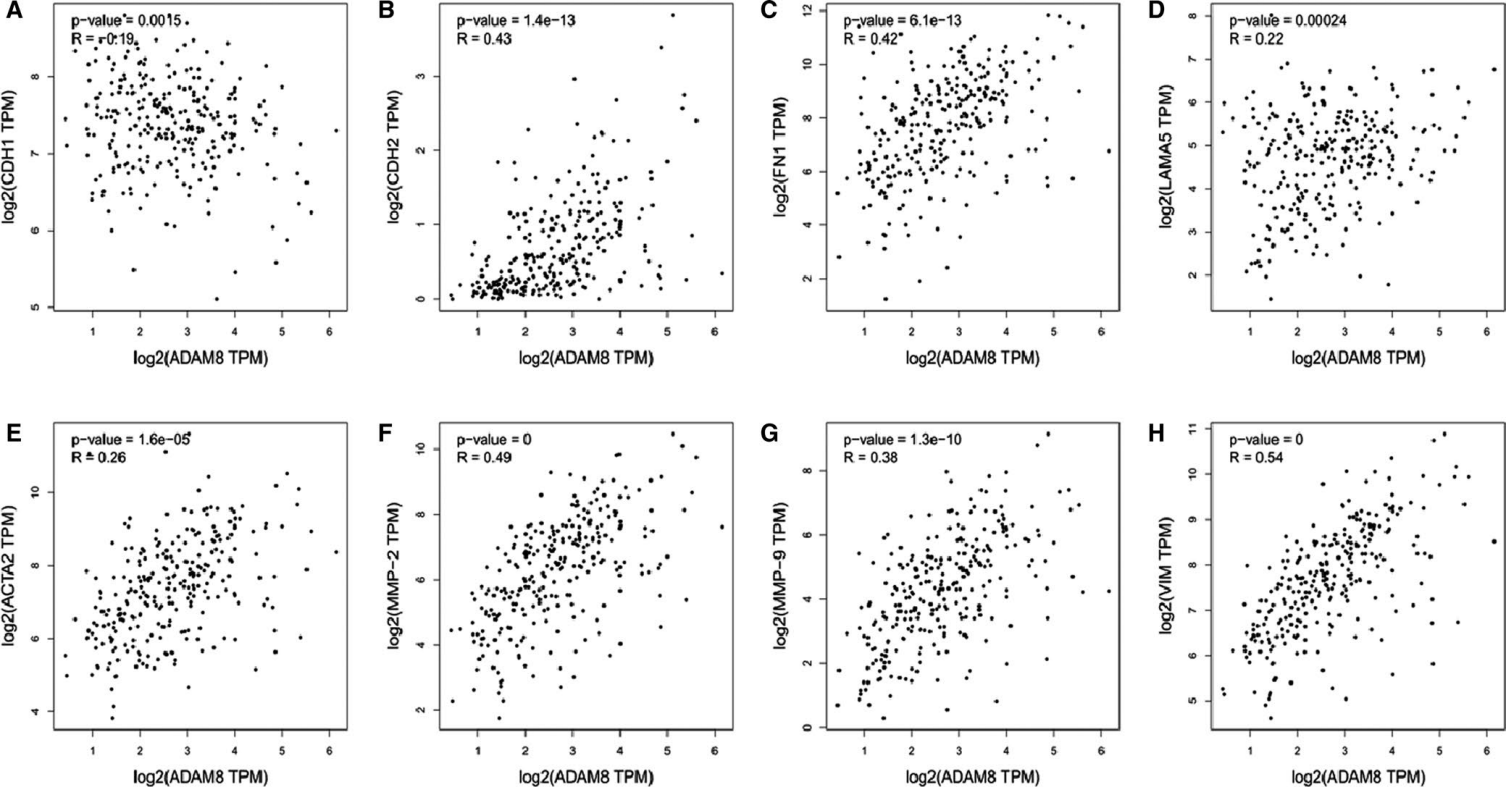
ADAM8 was correlated with multiple EMT biomarkers. The mRNA‐seq data were retrieved from the TCGA and GTEx database. Pearson correlation analysis was employed to determine the correlation between ADAM8 and these EMT biomarkers. A, E‐cadherin (CDH1), B, N‐cadherin (CDH2), C, fibronectin, D, Laminin, E, α‐SMA (ACTA2), F, MMP2, G, MMP‐9 and H, Vimentin. R: Pearson correlation coefficient. *P < .05; **P < .01; ***P < .001; ns, no significant

**Figure 3:**
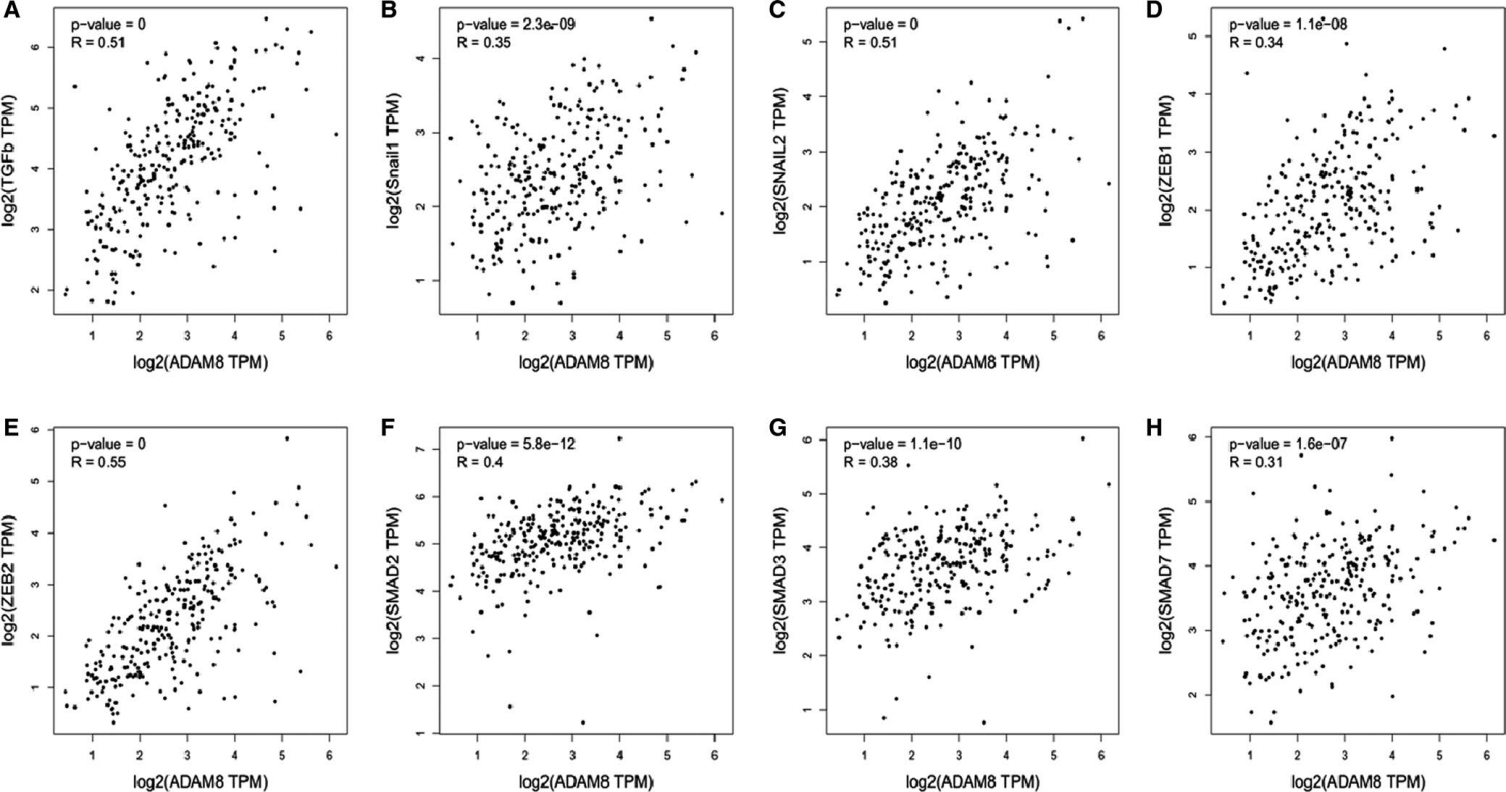
ADAM8 was correlated with multiple EMT‐related transcription factors. The mRNA‐seq data were retrieved from TCGA and GTEx database. The Pearson correlation analysis was employed to determine the correlation between ADAM8 and these EMT‐related transcription factors. A, TGF‐β, B, Snail1, C, Snail2, D, ZEB1, E, ZEB2, F, Smad2, G, Smad3 and H, Smad7. R: Pearson correlation coefficient. *P < .05; **P < .01; ***P < .001; ns, no significant

To determine whether ADAM8 associated with EMT in colon cancer cells, GEPIA was utilized to perform paired gene Pearson correlation analysis between ADAM8 and EMT‐related biomarkers. The results of Pearson correlation analysis showed that ADAM8 was associated with various EMT biomarkers (*P* < .05).^18^ These markers included cellular transmembrane proteins which included E‐cadherin (CDH1) and N‐cadherin (CDH2), cytoskeletal markers which included α‐SMA (ACTA2) and Vimentin and extracellular matrix (ECM) proteins (eg MMP‐2, MMP‐9, fibronectin and Laminin; Figure 2). ADAM8 was also linked to TGF‐β and some EMT‐related transcription factors (Smad2, Smad3, Smad7, Snail1, Snail2, ZEB1 and ZEB2; *P* < .05), especially TGF‐β, Snail 2 and Zeb2 with a moderate or higher correlation (*R* > 0.5, Figure 3). E‐cadherin (CDH1) was a factor which negatively correlated with ADAM8 (*P* = .0015. *R* = −0.19), while others were positively correlated with ADAM8. These results suggested that ADAM8 might play a pivotal role in EMT.

### 3.3 Up‐regulation of ADAM8 induced EMT and enhanced the invasive ability of colon cancer cells

**Figure 4:**
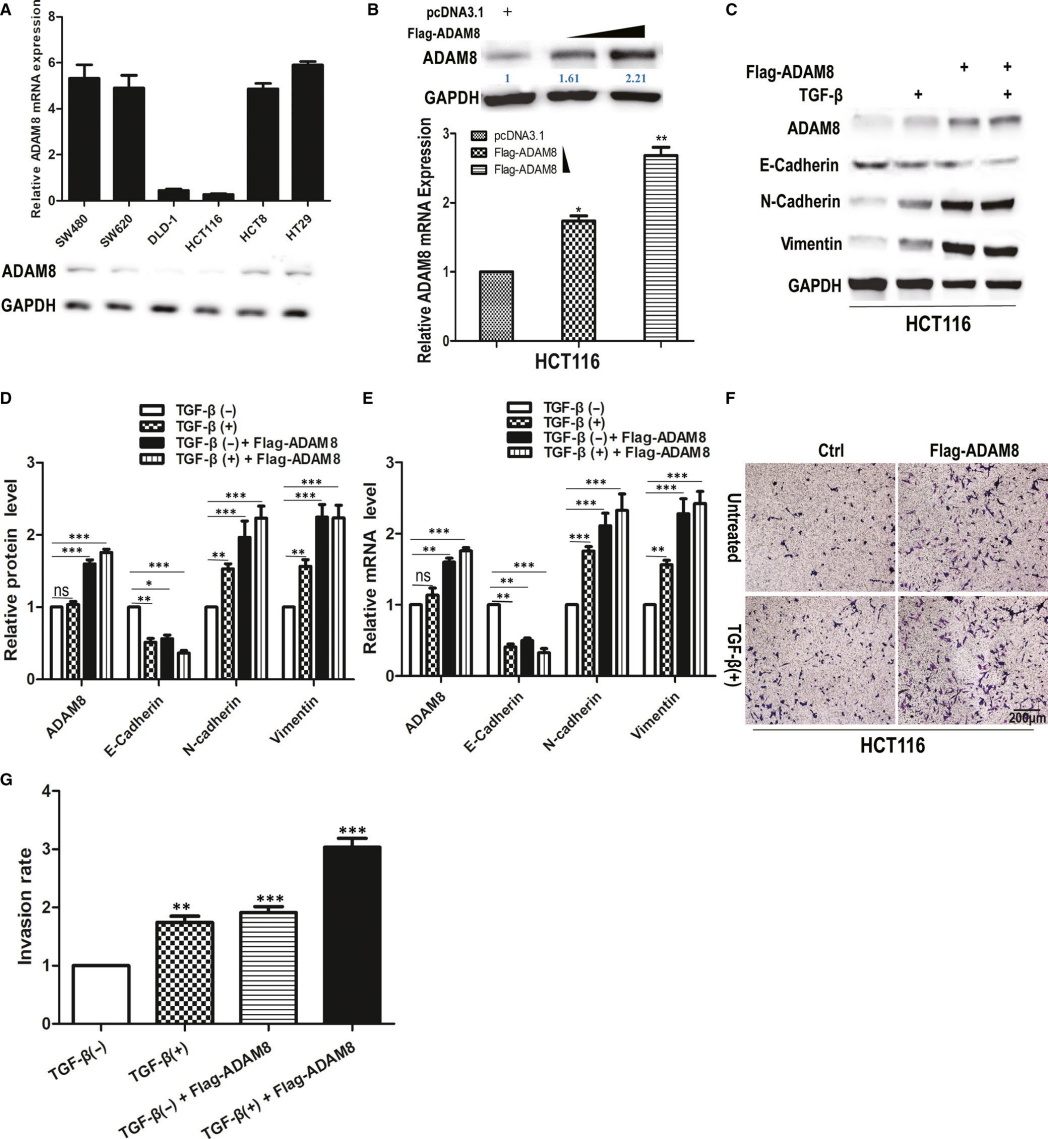
Up‐regulating ADAM8 induced EMT effect and enhanced the invasive ability of colon cancer cells. A, The level of ADAM8 at mRNA and protein in six colon cancer cell lines (SW480, SW620, DLD1, HCT116, HCT8 and HT29) was analysed by qRT‐PCR and Western blotting. B, HCT116 was transfected pcDNA3.1, 3 μg or 5 μg Flag‐ADAM8 for 48 h and cells were harvested for Western blotting and qRT‐PCR analysis. C, D and E, HCT116 cells treated with 5 μg Flag‐ADAM8 and 5 ng/mL TGF‐β for 48 h. Western blotting and qRT‐PCR were employed to assess the level of ADAM8, E‐cadherin, N‐cadherin, and Vimentin at protein and mRNA. F and G, Transwell invasion assay was used to assess the invasiveness of HCT116 treated with Flag‐ADAM8 and TGF‐β for 48 h. The average number of cells invaded through the filter was counted. The data were obtained from three independent experiments. The data were presented as the mean ± standard deviation and two‐way ANOVA. The expression of all proteins was quantified by Image J software and normalized to the quantified value of GAPDH. *P < .05; **P < .01; ***P < .001; ns, no significant

In the analysis about public database, it was shown that ADAM8 associated with various EMT biomarkers in colon cancer. To find more convincing evidence, we further verified the interaction between ADAM8 and EMT in colon cancer cells through in vitro experiments. Firstly, qRT‐PCR and Western blotting were utilized to detect the mRNA and protein level of ADAM8 in six common colon cancer cell lines (Figure 4A). Among six colon cancer cell lines, SW480 with higher endogenous expression and HCT116 with lower endogenous expression were selected for next‐step study. HCT116 with lower endogenous expression acted as a vector which transfected with ADAM8 overexpression plasmid (Flag‐ADAM8) to generate cell lines stably expressing ADAM8 (Figure 4B). It was known that TGF‐β was a potent inducer of EMT in various cancer epithelial cells.^19^ To observe the EMT in colon cancer cells, HCT116 was treated with 5 ng/mL TGF‐β or/and ADAM8 overexpression plasmid (Flag‐ADAM8) for 48 hours. Western blotting and qRT‐PCR were used to detect the protein and mRNA level of EMT biomarkers, respectively. The results of Western blotting showed that TGF‐β treatment significantly inhibited the expression of E‐cadherin (95% CI of diff. −0.9335~−0.03988, *P* < .01) but induced the expression of Vimentin (95% CI of diff. 0.1199 ~ 1.013, *P* < .01) and N‐cadherin (95% CI of diff. 0.08321 ~ 0.9768, *P* < .01) in colon cancer cells (Figure 4C,D). Besides, the ectopic overexpression of ADAM8 also decreased the expression of E‐cadherin protein (95% CI of diff. −0.8835 ~ 0.01012, *P* < .05), but increased the expression of Vimentin (95% CI of diff. 0.7999 ~ 1.693, *P* < .001) and N‐cadherin (95% CI of diff. 0.5199 ~ 1.413, *P* < .001; Figure 4C,D). The outcomes of qRT‐PCR further verified the above findings (Figure 4E). Moreover, Transwell assay was used to evaluate the effect of ADAM8 on the invasion of cancer cells. As a result, the invasive ability of HCT116 was enhanced when treated with TGF‐β (*F* = 44.59, *P* = .0026) and ADAM8 overexpression plasmid (Flag‐ADAM8; *F* = 78.12, *P* = .009; Figure 4F,G). Therefore, combined with the results of Pearson correlation analysis, ADAM8 was an important factor for inducing EMT in colon cancer cells. The up‐regulation of ADAM8 could induce EMT phenotype and enhance the invasive ability of colon cancer cells.

### 3.4 Down‐regulation of ADAM8 attenuated EMT and colon cancer cell invasion

**Figure 5:**
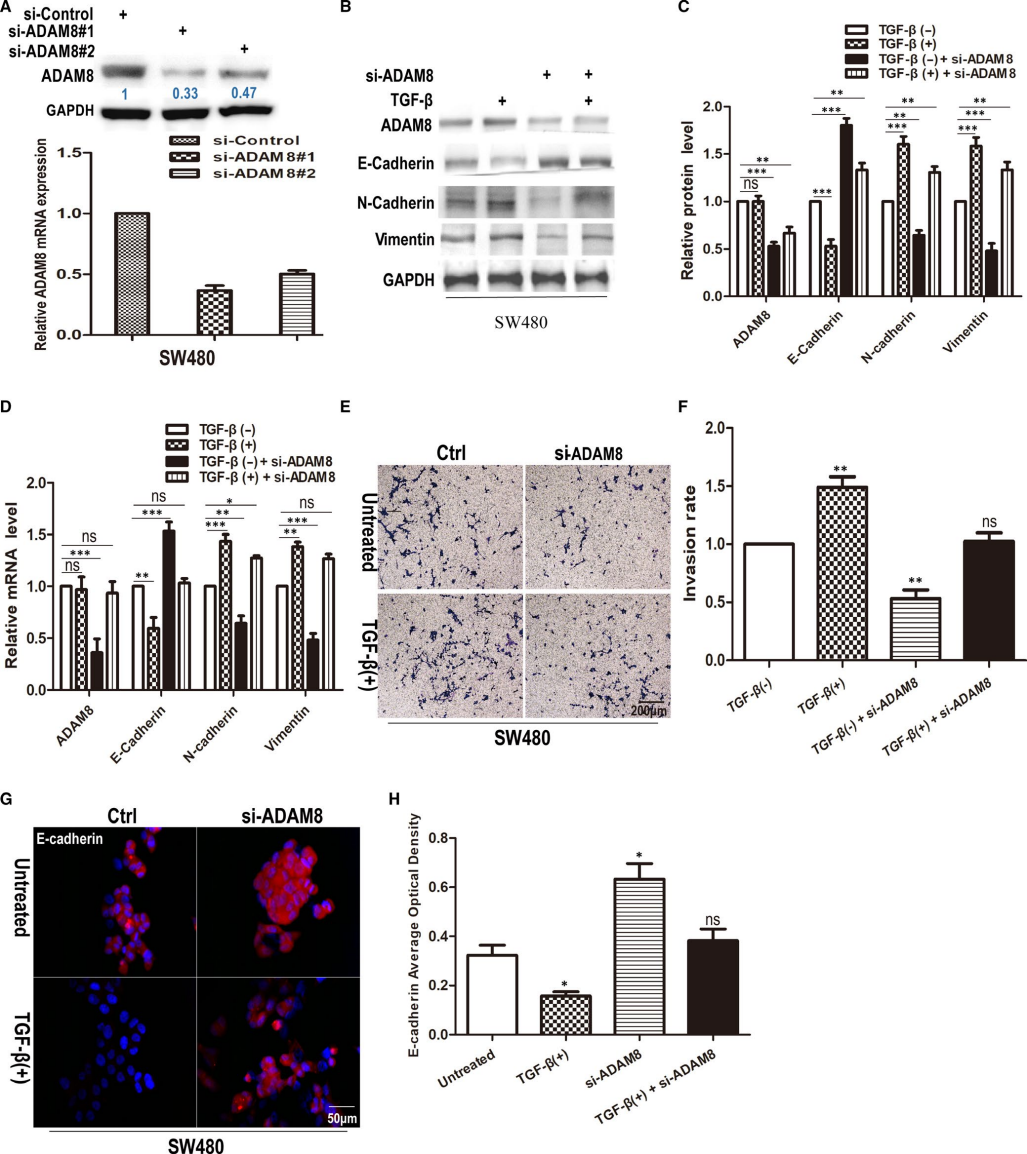
Down‐regulating ADAM8 attenuated EMT effect and colon cancer cell invasion. A, SW480 was transfected with si‐Control, si‐ADAM8#1 or #2 for 48 h. Cells were harvested for Western blotting and qRT‐PCR analysis. B, C and D, SW480 cells treated with si‐ADAM8 and 5 ng/mL TGF‐β for 48 h. Western blotting and qRT‐PCR were employed to assess the level of ADAM8, E‐cadherin, N‐cadherin and Vimentin at protein and mRNA. (E) and (F) Transwell invasion assay was employed to assess the invasiveness of SW480 treated with si‐ADAM8 and TGF‐β for 48 h. The average number of cells invaded through the filter was counted. (G) and (H) Immunofluorescence staining was used to assess the expression of E‐cadherin. E‐cadherin was red, and the nuclei of the cells were blue. The data were obtained from three independent experiments. The data were presented as the mean ± standard deviation and two‐way ANOVA. The expression of all proteins was quantified by Image J software and normalized to the quantified value of GAPDH. *P < .05; **P < .01; ***P < .001; ns, no significant

SW480 with higher endogenous ADAM8 expression served as a vector which transfected with the siRNA for ADAM8 (si‐ADAM8). The results of Western blotting and qRT‐PCR indicated that si‐ADAM8#1, which could inhibit the expression of ADAM8 greater than si‐ADAM8#2, was more suitable for next‐step experiments (Figure 5A). To observe the EMT of colon cancer cells, SW480 was treated with si‐ADAM8 or/and 5 ng/mL TGF‐β for 48 hours. Western blotting and qRT‐PCR were utilized to test the protein and mRNA level of EMT biomarkers, respectively. The results of Western blotting showed that TGF‐β treatment significantly inhibited E‐cadherin expression in SW480 (95% CI of diff. −0.7377~−0.20238, *P* < .001) and enhanced the expression of Vimentin (95% CI of diff. 0.3156 ~ 0.8510, *P* < .001) and N‐cadherin (95% CI of diff. 0.3356 ~ 0.8710, *P* < .001; Figure 5B,C). Moreover, the down‐regulation of ADAM8 could reduce the expression of Vimentin (95% CI of diff. −0.7877~−0.2523, *P* < .001) and N‐cadherin (95% CI of diff. −0.6244~−0.08897, *P* < .01) in SW480 cells. Meanwhile, the expression of E‐cadherin was enhanced (95% CI of diff. 0.5356 ~ 1.071, *P* < .001; Figure 5B,C) after down‐regulating the expression of ADAM8. The similar result was achieved by qRT‐PCR (Figure 5D). Using Transwell invasion assay to assess the effect of ADAM8 on the invasion of tumour cells, knockdown of ADAM8 with siRNA significantly reduced the invasion of colon cancer cells (*F* = 42.38, *P* = .0029). More importantly, down‐regulation of ADAM8 could attenuate TGF‐β‐mediated cell invasion (*F* = 0.14, *P* = .72) (Figure 5E,F

). The result of immunofluorescence also showed that the expression of E‐cadherin in SW480 with si‐ADAM8 was significantly enhanced, and the down‐regulation of ADAM8 could attenuate the TGF‐β‐mediated inhibition of E‐cadherin expression. (Figure 5G,H). In brief, these results indicated that the down‐regulation of ADAM8 attenuated EMT and colon cancer cell invasion.

### 3.5 ADAM8 modulated EMT by activating TGF‐β/Smad2/3 signalling pathway

**Figure 6:**
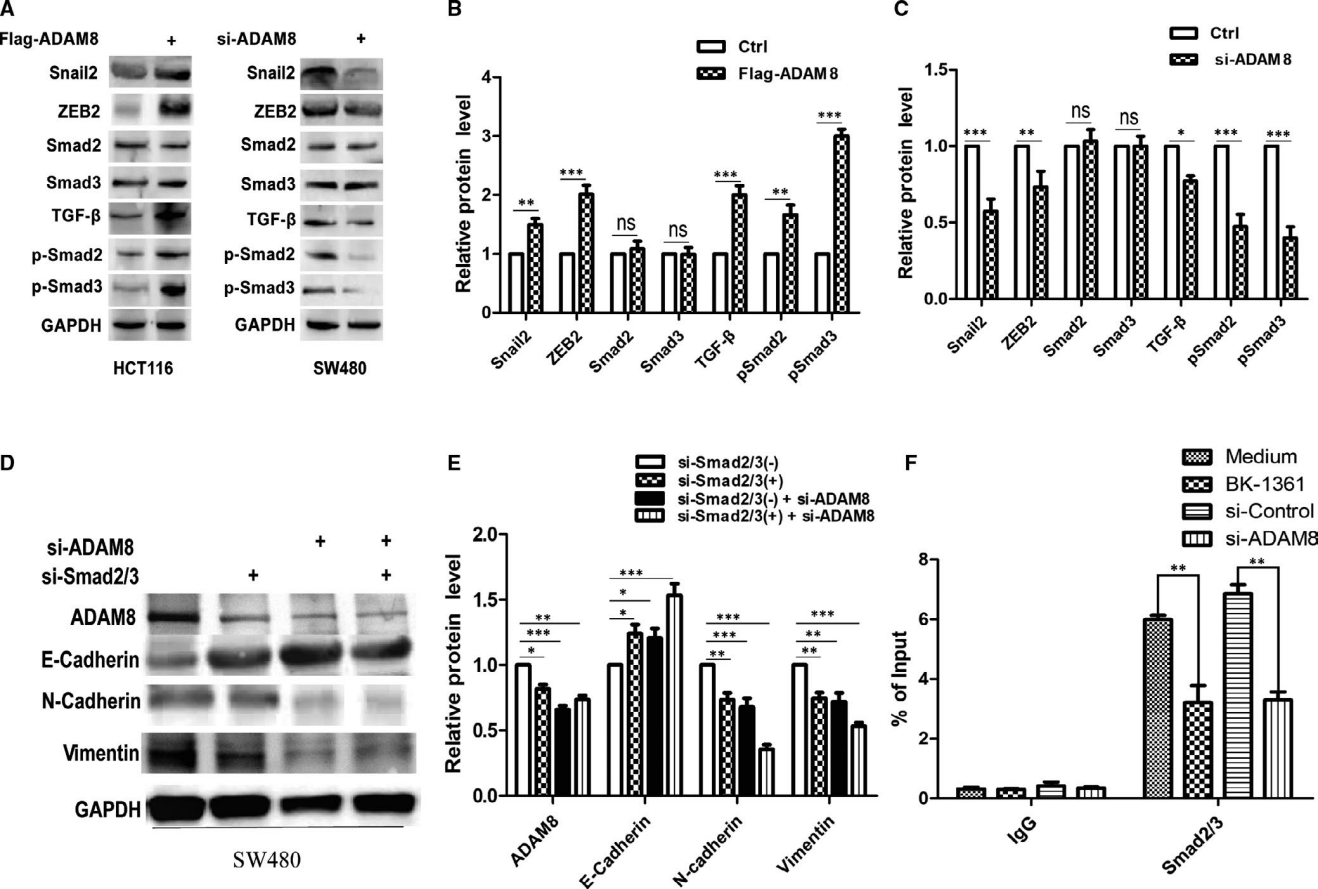
ADAM8 modulated the EMT via activating TGF‐β/Smad2/3 signalling pathway. A, B and C, Colon cancer cells were treated with si‐ADAM8 or Flag‐ADAM8 for 48 h. Western blotting was employed to assess the level of Snail2, ZEB2, TGF‐β, Smad2, pSmad2, Smad3 and pSmad3 protein. D and E, SW480 cells were treated with si‐ADAM8 and si‐Smad2/3 for 48 h. Western blotting analysis was used to assess the expression of ADAM8, E‐cadherin, N‐cadherin and Vimentin protein. F, ChIP‐qPCR analysis of Smad2/3 binding at the E‐cadherin gene promoter in SW480 after treated with Medium or BK‐1361 (5 µmol/L) for 24 h or infected with indicated constructs for 48 h. The data were obtained from three independent experiments. The data were presented as the mean ± standard deviation and two‐way ANOVA. The expression of all proteins was quantified by Image J software and normalized to the quantified value of GAPDH. *P < .05; **P < .01; ***P < .001; ns, no significant

It was well known that TGF‐β could mediate the canonical Smad (small mothers against decapentaplegic) signalling pathway and the non‐canonical signalling pathway. The activation of canonical Smad signalling pathway induced the phosphorylation and activation of the type I TGF‐β receptor (TGFβRI) through TGF‐β binding to the type II TGF‐β receptor (TGFβRII). The activated TGFβRI phosphorylates Smad2 and Smad3, which then formed a heteromeric complex with Smad4 and accumulated into the nucleus to regulate transcription.^20^ To investigate whether Smad2/3 was a key mediator between ADAM8 and EMT, HCT116 and SW480 were, respectively, treated with Flag‐ADAM8 and si‐ADAM8, and Western blotting was employed to assess the expression levels of Snail2, ZEB2, TGF‐β, Smad2 and Smad3 protein. The results showed that the expression of Snail2, ZEB2, TGF‐β, pSmad2 and pSmad3 was significantly increased in HCT116 with ectopic ADAM8 expression (*P* < .05, Figure 6A,B), while knockdown of ADAM8 in SW480 cells diminished the expression of Snail2, ZEB2, TGF‐β, pSmad2 and pSmad3 (*P* < .05, Figure 6A,C). Moreover, knockdown of Smad2/3 in SW480 cells could inhibit the expression of Vimentin (95% CI of diff. −0.4848~−0.02316, *P* < .01) and N‐cadherin (95% CI of diff. −0.4975~−0.03583, *P* < .01), but enhance the expression of E‐cadherin (95% CI of diff. 0.009164 ~ 0.4708, *P* < .05; Figure 6D,E). Importantly, we demonstrated that the treatment with BK‐1361 or the knockdown of ADAM8 in SW480 cells attenuated the Smad2/3 binding to the promoter of E‐cadherin via using ChIP‐qPCR assay (Figure 6F). In a word, these results indicated that ADAM8 was involved in the regulation of EMT processes in colon cancer cells through activating TGF‐β/Smad2/3 signalling pathway.

## 4 DISCUSSION

EMT was considered to be the initial step of tumour cell invasion. It manifested in the loss of cell polarity and cell adhesion to obtain migration and invasiveness and played an important role in initiating and promoting tumour cell invasion and metastasis.^18^ EMT was associated with a series of molecular events which included down‐regulation and dysfunction of E‐cadherin, relocalization of β‐catenin from membrane to nucleus and up‐regulation of the mesenchymal marker proteins (eg N‐cadherin, Vimentin).^21^, ^22^ Besides, the expression of EMT‐related transcription factors, including Twist, Snail1, Snail2, Zeb1 and Zeb2, promoted the EMT processes. Its mechanisms encompassed reprogramming of tumour cells to obtain stem cell characteristics, leading to rapid tumour development and inefficient treatment, inhibiting tumour cell senescence and apoptosis and promoting tumour cells metabolic changes.^23^

As mentioned above, the level of ADAM8 was closely related to the invasion and metastasis of various malignant tumours and influenced the prognosis of cancer patients. In a recent review, Conrad *et al* ^24^ summarized five mechanisms in which ADAM8 involved in cancer progression as follows: forming the pro‐tumorigenic microenvironment, dissociating ECM components, producing chemical resistance and modulating the extracellular activity of MMPs (matrix metalloproteinases), regulating cell motility and adhesion and inducing the formation of new blood vessels. To the best of our knowledge, our study is the first to explore the specific mechanism by which ADAM8 promoted the invasion of colon cancer cells by inducing EMT.

The online tool GEPIA was utilized to analyse colon cancer patients’ data in the TCGA and GTEx databases. It was shown that the overall survival of colon cancer patients with high ADAM8 expression was significantly lower than that of patients with low ADAM8 expression. The result was consistent with another study in which Yang *et al* ^7^ concluded that patients with ADAM8‐positive tumours had worse 5‐year disease‐free survival and overall survival. More importantly, we discovered the link between ADAM8 and EMT biomarkers. ADAM8 with a multi‐structural working domain played an important role in extracellular proteolysis, such as releasing chemokines and cytokines via ectodomain shedding (eg CD23, TNF receptor 1 and IL‐1 receptor 2) and cleaving important ECM components of the tumour stroma (eg collagen I, fibronectin and periostin).^25^, ^26^ It was hypothesized that ADAM8 had an important effect on colon cancer EMT processes. Our experimental results validated the above hypothesis. It was shown that ADAM8 reduced the expression of epithelial markers (E‐cadherin) and increased the expression of mesenchymal markers (Vimentin and N‐cadherin), which indicated that ADAM8 could induce EMT in colon cancer cells. Moreover, we found that ADAM8 could promote the invasion of colon cancer cells and modulate EMT by TGF‐β/Smad2/3 signalling pathway.

It was well established that in addition to disintegrating and remodelling ECM, ADAM8 also involved in the activation of integrins through the so‐called ‘integrin‐binding loop’ and led to the migration and invasion of cancer cells. The membrane localization of ADAM8 in pancreatic cancer cells suggested that ADAM8 was complexed with β1 integrin thereby enhancing cell migration and invasiveness.^27^ Conrad *et al* ^28^ concluded that ADAM8 could change the adhesive properties of breast cancer MB‐231 cells to endothelia by influencing β1 integrin cell surface localization and activation thereby changing the cell morphology into an invasive phenotype. The result of Romagnoli's study showed that ADAM8 promoted breast tumour cell adhesion onto the endothelium and dissemination via β1‐integrin activation in vivo.^8^ Apart from acting as an inducer of EMT, TGF‐β was also a key regulator of ECM production, deposition and remodelling.^29^ In EMT, perturbations of the ECM microenvironment or intracellular cues could persuade integrins to dictate adhesion changes between cells and the ECM, subsequently inducing disassembly of tight and adherens junctions, dissolution of desmosomes, actin reorganization and loss of epithelial cell polarity. These changes initiated focal adhesion complex formation leading to cancer cell migration and invasion.^30^ Furthermore, integrins could facilitate changes in cell‐ECM contact during EMT to control their function.^31^ β1 integrin engagement with collagen type I resulted in the loss of E‐cadherin and indirect up‐regulation of N‐cadherin, suggesting that integrin activation could directly result in EMT.^32^, ^33^ β5 integrin regulated cell‐cell/cell‐ECM adhesion changes upon TGF‐β signalling and β5 integrin depletion reduced the invasiveness of breast carcinoma cells by impairing the dissociation of tight junctions and/or reducing cell–ECM adhesion.^34^, ^35^ Integrins and their signalling could regulate EMT by perturbing cell adhesion and stimulating EMT‐associated gene expression. Integrins could activate latent TGF‐β to result in TGF‐β‐induced EMT.^35^, ^36^ Our research also found that up‐regulation of ADAM8 could enhance the expression of TGF‐β, while down‐regulation of ADAM8 diminished the expression of TGF‐β. Based on previous studies and our findings, ADAM8 should be the upstream factor of TGF‐β. The possible mechanism might be ADAM8 activated a certain integrin, leading to the release of latent TGF‐β in colon cancer. In addition, Pickup *et al* ^37^ found that TGF‐β induced EMT via Smad2/3 signalling pathway. This canonical pathway could also activate integrin‐linked kinase (ILK), thereby phosphorylate GSK‐3β and Akt, leading to β‐catenin nuclear translocation and activation of other transcription factors, which induce EMT of epithelial cells.^38^, ^39^ Thus, ADAM8 induced EMT in colon cancer cells through TGF‐β/Smad2/3 signalling pathway, and integrins were the key mediators between the interaction of ADAM8 and TGF‐β.

In conclusion, our study indicated that ADAM8 was an important biomarker for the prognosis of colon cancer and induced EMT to promote the invasion of colon cancer cells via activating TGF‐β/Smad2/3 signalling pathway. The present study elucidated the specific mechanism of ADAM8 promoting colon cancer cell invasion and laid the groundwork for future research of ADAM8 as a promising therapeutic target for colon cancer.

## CONFLICT OF INTEREST

The authors have no conflict of interest to declare.

## AUTHOR CONTRIBUTION

**Qianna Jin:** Conceptualization (equal); Data curation (equal); Investigation (equal); Methodology (equal); Writing‐original draft (equal); Writing‐review & editing (equal). **Xin Jin:** Investigation (equal); Methodology (equal). **Tao Liu:** Resources (equal). **Xiaoming Lu:** Resources (equal). **Guobin Wang:** Funding acquisition (equal); Project administration (equal); Supervision (equal). **Nan He:** Conceptualization (lead); Data curation (lead); Formal analysis (lead); Funding acquisition (equal); Investigation (equal); Methodology (equal); Project administration (lead); Software (lead); Supervision (equal); Writing‐original draft (lead); Writing‐review & editing (lead).

## Data Availability

The data sets used in the current study are available from the corresponding author on reasonable request.
